# Analysis of timeliness of infectious disease reporting in the Netherlands

**DOI:** 10.1186/1471-2458-11-409

**Published:** 2011-05-30

**Authors:** Elisabeth Reijn, Corien M Swaan, Mirjam EE Kretzschmar, Jim E van Steenbergen

**Affiliations:** 1 Municipal Health Service (MHS) Zaanstreek-Waterland, Zaandam, the Netherlands; 2 Preparedness and Response Unit; Centre for Infectious Disease Control; National Institute for Public Health and the Environment (RIVM), Bilthoven, the Netherlands; 3 Julius Centre for Health Sciences & Primary Care; University Medical Centre Utrecht, Utrecht, the Netherlands; 4 Epidemiology and Surveillance Unit; Centre for Infectious Disease Control; National Institute for Public Health and the Environment (RIVM), Bilthoven, the Netherlands; 5 Centre for Infectious Diseases; Leiden University Medical Centre, Leiden, the Netherlands

## Abstract

**Background:**

Timely reporting of infectious disease cases to public health authorities is essential to effective public health response. To evaluate the timeliness of reporting to the Dutch Municipal Health Services (MHS), we used as quantitative measures the intervals between onset of symptoms and MHS notification, and between laboratory diagnosis and notification with regard to six notifiable diseases.

**Methods:**

We retrieved reporting data from June 2003 to December 2008 from the Dutch national notification system for shigellosis, EHEC/STEC infection, typhoid fever, measles, meningococcal disease, and hepatitis A virus (HAV) infection. For each disease, median intervals between date of onset and MHS notification were calculated and compared with the median incubation period. The median interval between date of laboratory diagnosis and MHS notification was similarly analysed. For the year 2008, we also investigated whether timeliness is improved by MHS agreements with physicians and laboratories that allow direct laboratory reporting. Finally, we investigated whether reports made by post, fax, or e-mail were more timely.

**Results:**

The percentage of infectious diseases reported within one incubation period varied widely, between 0.4% for shigellosis and 90.3% for HAV infection. Not reported within two incubation periods were 97.1% of shigellosis cases, 76.2% of cases of EHEC/STEC infection, 13.3% of meningococcosis cases, 15.7% of measles cases, and 29.7% of typhoid fever cases. A substantial percentage of infectious disease cases was reported more than three days after laboratory diagnosis, varying between 12% for meningococcosis and 42% for shigellosis. MHS which had agreements with physicians and laboratories showed a significantly shorter notification time compared to MHS without such agreements.

**Conclusions:**

Over the study period, many cases of the six notifiable diseases were not reported within two incubation periods, and many were reported more than three days after laboratory diagnosis. An increase in direct laboratory reporting of diagnoses to MHS would improve timeliness, as would the use of fax rather than post or e-mail. Automated reporting systems have to be explored in the Netherlands. Development of standardised and improved measures for timeliness is needed.

## Background

Accurate communicable disease surveillance systems are essential to initiate and sustain effective public health response and control measures. Efforts to improve completeness and timeliness of surveillance data on infectious diseases must therefore be part of a continuous process to improve the quality of surveillance systems in order to minimise further spread of disease [1-3].

Timeliness in reporting is crucial in preventing secondary cases and outbreaks of infectious diseases. Delays in the notification process can occur in patients going to their general practitioner or in reporting the laboratory diagnosis to the MHS. According to guidelines, produced by the United States Centers for Disease Control and Prevention, timeliness should be periodically evaluated for each specific surveillance step of each notifiable disease [4]. Nonetheless, a quantitative measure of timeliness of surveillance systems has not been standardised, and study methods vary, often comparing conventional paper-based systems to electronic systems [5,6] or referring to time limits that differ among countries [7].

Studies with comparable data on surveillance intervals and timeliness are few in number [6-9]. Some surveillance systems did not always record date of symptom onset of disease; some analysed time intervals that were omitted by others, such as the period between hospital admission and notification date [9,10].

The incubation period of a disease, as a proxy for its period of transmissibility, has been used as a quantitative measure to evaluate timeliness of reporting [6,8]. This is justified, as it relates to the time before secondary cases may occur and is also the most effective time for prevention and control measures, such as active or passive immunisation of close contacts or the use of post-exposure prophylaxis [6,11]. In addition, outbreaks are often assumed to have ended after two incubation periods have passed since the end of the period of infectiousness of the last case.

In the Netherlands, physicians are legally obliged to report laboratory-confirmed diagnoses of certain communicable diseases to the local Municipal Health Service (MHS). They should report within one day or within three days if a weekend intervenes. In turn, the MHS reports overnight to the National Institute for Public Health and the Environment (RIVM), using a web-based application [5].

The introduction of mandatory reporting by laboratories as well as by physicians could potentially improve timeliness [12-15]. During our study period, this hypothesis was investigated by examining physician-laboratory-MHS agreements that authorised direct reporting by one or more local laboratories.

Timeliness might also be influenced by the method of reporting. Traditionally, physicians used paper "report cards" provided by the Inspectorate of Healthcare as a postal notification form, but these are little used nowadays. A minority of laboratories and physicians use electronic(e)-mail for reporting to the MHS; the precise proportion is unknown.

In this study we performed a quantitative analysis of the timeliness of infectious disease reporting in the Netherlands for six diseases. The intervals between onset of symptoms and MHS notification, and between laboratory diagnosis and notification, were used as quantitative measures of timeliness. These intervals can be influenced in different ways. Public health authorities can raise alertness of patients and physicians with regard to certain diseases so that laboratory tests are requested at an early stage. After laboratory confirmation of diagnoses, reporting is most influenced by delays in communication. Such delays should be avoided, and so this interval is separately analysed in our study.

We report here serious reporting delays per disease, expressed in the percentage of notifications occurring within one and two incubation periods.

We relate timeliness to the existence of physician-laboratory-MHS agreements, different methods of reporting (post, fax, telephone, e-mail), and the number of notifications using "report cards."

## Methods

We selected notifiable infectious diseases based on their epidemic potential, number of reported cases, use of laboratory confirmation, and available literature for comparisons. As a result, reports for six infectious diseases were studied: shigellosis, entero-haemorrhagic or Shiga-like toxin-producing E. coli (EHEC/STEC) infection, typhoid fever, measles, meningococcosis, and hepatitis A virus (HAV) infection. The criteria for reporting these infectious diseases in the Netherlands are the clinical criteria in combination with the confirmation of the diagnosis by laboratory testing. Notifications from June 2003 to December 2008 were retrieved from the Dutch national database, including date of symptom onset, date of laboratory-confirmed diagnosis, and date of reporting to the Municipal Health Service (MHS).

**Figure 1:**
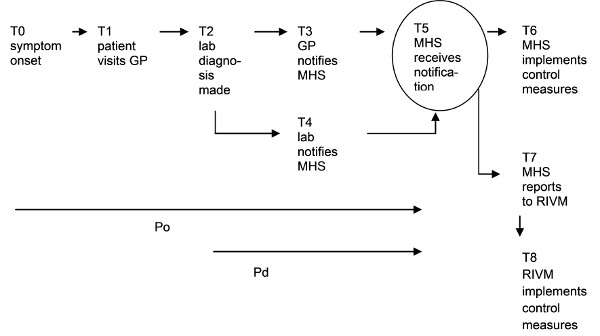
**Time-points (T) and intervals (P) in the notification process**. GP = general practitioner; Po = period between symptom onset and MHS notification; Pd = period between laboratory diagnosis and MHS notification; MHS = Municipal Health Service; RIVM = National Institute for Public Health and the Environment.

For each case, we determined the intervals between the zero timepoint of symptom onset (T0) and MHS notification (T5), and between laboratory diagnosis (T2) and MHS notification (T5). As shown in Figure 1, the interval between timepoints onset (T0) and notification (T5) was defined as the period from onset of symptoms until notification (Po); the interval between timepoints diagnosis (T2) and notification (T5) was defined as the period from laboratory diagnosis until notification (Pd). We then determined the median of the distributions of Po and Pd for each of the six diseases.

For comparison with the notification data, we used the midpoint of the range of the incubation period for each disease, as it is defined in the literature and in the national guidelines of the RIVM in the Netherlands [16]. We then calculated the percentage of notifications per disease occurring within one and two incubation periods respectively (in the context of preventing the occurrence of tertiary cases).

**Figure 2:**
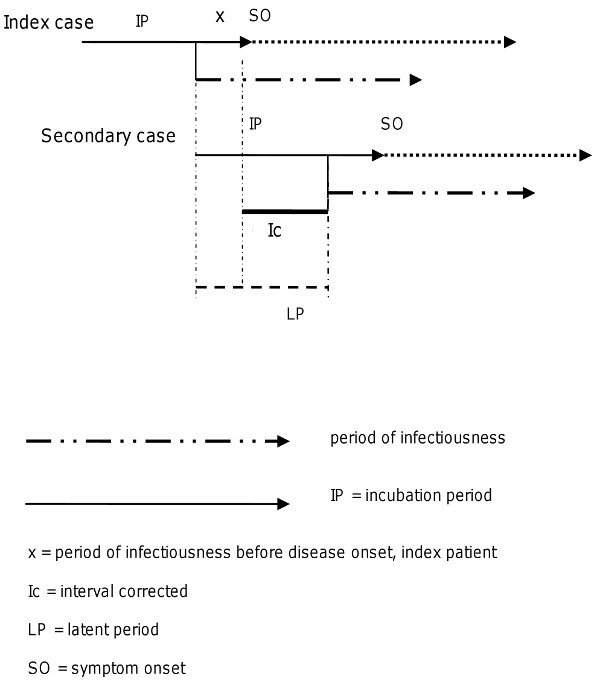
**Ic (interval corrected) = LP (latent period) - × (period of infectiousness before disease onset, index patient)**.

There is international consensus that for HAV infections and measles, the period of infectiousness starts before onset of symptomatic disease. The incubation period (IP) for these diseases is thus less valid as a proxy for the period in which public health measures are most effective. More adequate for these diseases is the latent period (LP), defined as the period between being infected and becoming infectious for others [17]. Reporting of the index case should preferably occur before the onset of infectiousness in a possible secondary case. As a secondary case can be infected before symptom onset in the index patient, this time interval must be subtracted from the LP. We therefore defined a corrected time interval (Ic) as the interval from symptom onset in the index case to the start of infectiousness in one or more secondary cases. As shown in Figure 2: Ic (interval corrected) = LP (latent period) - × (period of infectiousness in index patient preceding symptom onset)

For HAV infections, × is about seven days; for measles, × is 1-2 days. Thus the Ic for HAV infection is 14 days, given the LP of 21 days. The Ic for measles is seven days, given the LP of 8-9 days.

Of the six diseases under study, shigellosis and HAV infection were used in the calculation of distribution means of Po and Pd per year, in order to identify possible trends or changes over the years.

The percentage of notifications occurring more than three days after laboratory diagnosis was calculated per disease. We devised a hypothetical scenario with an interval Pd of zero to demonstrate the improvement made possible by immediate reporting (on the day of laboratory diagnosis), using fax, telephone, or e-mail.

All MHS (n = 31) in the Netherlands participated and were sent a questionnaire regarding timeliness and methods of reporting hepatitis B virus (HBV) infection in the year 2008. This disease was chosen because, unlike many of the other six, it is reported frequently enough over the years, that data could be expected from all MHS in all parts of the country.

The questionnaire asked whether physician-laboratory-MHS agreements were in place to allow direct reporting of cases of HBV infections by laboratories. It inquired about methods of reporting (post/fax/phone/e-mail) and the numbers of "report cards" received by post each week. The MHS respondents whose questionnaires mentioned experience with direct laboratory reporting by e-mail were interviewed by telephone about the number of these reports, the speed of reporting and the security of their inhouse electronic mail system.

All analyses were performed in SPSS version 17. Statistical analysis was performed using Independent Samples T-test and One-Way analysis of variance (One-Way ANOVA, post hoc Multiple Comparisons).

## Results

### Time intervals Po and Pd

In the study period we analysed 1910 reports on shigellosis, 432 reports on EHEC/STEC infection, 1263 reports on meningococcosis, 134 reports on measles, 166 reports on typhoid fever and 1518 reports on HAV infection. Some reports had missing or incorrect data for the onset of symptoms timepoint and were therefore excluded. The median period from onset until notification (Po) varied between three days for meningococcosis to 16 days for typhoid fever (Table 1). When comparing the disease-specific incubation periods, the median intervals for shigellosis, EHEC/STEC infection, and typhoid fever exceeded the midpoints of the incubation periods by several days, whereas median delays of meningococcosis and measles were nearly equal to the incubation periods. The percentage of notifications that occurred within one incubation period varied widely, between 0.4% for shigellosis and 90.3% for HAV infection. Not reported within two incubation periods were 97.1% of shigellosis cases, 76.2% of cases of EHEC/STEC infection, 13.3% of meningococcosis cases, 15.7% of measles cases, and 29.7% of typhoid fever cases.

**Table 1:** Reporting time interval from symptom onset and from diagnosis until notification (Po and Pd) for infectious disease notifications in relation to the incubation period and in relation to the time after diagnosis, with the values assuming zero delay in laboratory reporting (underlined figures in italics)

	median Po (days)		percentage notifications ≤ 1IP (%)		percentage notifications ≤ 2IP (%)		m	median Pd (days)	percentage notifications >3days after diagnosis (%)	m
Shigellosis (IP 2 days) n=1910	**15**	***11***	0.4	*5.9*	2.9	*12.9*	5	**3**	42	0
EHEC/STEC (IP 3-4 days) n=432	**12**	***8***	2.3	*10.6*	23.8	*43.2*	49	**1**	33.3	11
										
Meningococcosis (IP 3-4 days) n=1263	**3**	***2***	57.1	*71.2*	86.7	*94*	19	**0**	12	2
										
Measles (IP 10 days) n=134	**8**	***6***	67.2	*71.6*	84.3	*85.8*	0	**0**	15.7	0
										
Typhoid fever (IP 11 days) n=166	**16**	***12***	33.5	*47.5*	70.3	*77.2*	8	**2**	22.3	0
										
HAV infection (IP 28 days) n=1518	**9**	***8***	90.3	*93.1*	97.2	*98*	65	**0**	20.9	1

Po = period from symptom onset until notification

Pd = period from laboratory diagnosis until notification

IP = incubation period

n = total number of notifications

m = number of missing i.e. excluded notifications

In contrast, given the long incubation period of HAV infections, only 2.8% of cases of HAV infection were not reported within two incubation periods. However, having corrected for the period of infectiousness before disease onset (Ic), the percentage of HAV infection notifications within one and two corrected intervals was substantially lower, with 71.4% of cases reported within one Ic (Table 2). Likewise for measles cases, the percentage reported within one Ic was lower (48.5%) than reported within one incubation period (67.2%).

**Table 2:** Percentage of notifications within one and two intervals corrected for the period of infectiousness before disease onset (Ic) for measles and hepatitis A virus infections, with the percentage assuming zero delay in laboratory reporting (underlined percentages in italics)

	percentage notifications ≤ 1 Ic (%)		percentage notifications ≤ 2 Ic (%)		m
Measles (Ic 7 days) n=134	48.5	*60.4*	71.6	*73.9*	0
					
HAV infection (Ic 14 days) n=1518	71.4	*78.7*	90.3	*93.1*	65

Ic (interval corrected) = LP (latent period)-× (period of infectiousness in index patient preceding symptom onset)

[ ]= total number of notifications

m = number of missing i.e. excluded notifications

For all six diseases, the median distribution of the period from diagnosis until notification (Pd) was between zero and three days, but a substantial percentage of notifications occurred more than three days after diagnosis, varying between 12% for meningococcosis and 42% for shigellosis. Assuming a Pd of zero (i.e. MHS notification on the same day as laboratory diagnosis), the median Po decreased by one day for meningococcosis and HAV infection to as much as four days for shigellosis, EHEC/STEC infection, and typhoid fever (underlined figures in italics in Table 1).

For meningococcosis, the percentage of notifications occurring within one incubation period would be 14% higher if notification were to take place on the day of laboratory diagnosis. For shigellosis and EHEC/STEC infections, the improvement was likewise substantial (5.5% and 8.3% within one incubation period and 10% and 19.4% within two incubation periods). However, the overall percentage of cases reported within one incubation period remained low, at 5.9% and 10.6%, respectively.

### Time trends

For shigellosis and HAV infection, the mean Pd decreased by one day from 2003 and 2004 to 2008, the period of our study. For HAV infection this decrease is not statistically significant. The mean Po for shigellosis and HAV infections however, showed no clear time-trend over the years (Table 3).

**Table 3:** Mean reporting time intervals from symptom onset and from diagnosis until notification (Po and Pd) for infectious disease notifications per year for shigellosis and hepatitis A virus infection

	Shigellosis						Hepatitis A virus infection					
	
	Po (days )	95% CI	n	Pd (days)	95% CI	n	Po (days)	95% CI	n	Pd (days)	95% CI	n
2003	**21.2**	18.2-24.1	*189*	**4.4**	3.6-5.2	*189*	**12.5**	11.1-13.8	*249*	**2.7**	2.1-3.3	*258*
2004	**21.7**	18.9-24.6	*336*	**4.5**	3.8-5.1	*339*	**14.5**	12.3-16.6	*420*	**3.8**	2.4-5.1	*439*
2005	**20.0**	17.9-22.1	*388*	**4.0**	3.4-4.7	*388*	**15.2**	12.2-18.2	*202*	**3.0**	1.5-4.6	*212*
2006	**22.2**	19.0-25.4	*238*	**3.8**	3.1-4.5	*239*	**15.1**	13.2-16.9	*261*	**2.4**	1.5-3.3	*277*
2007	**19.9**	18.1-21.6	*382*	**4.1**	3.4-4.8	*383*	**13.2**	11.1-15.3	*153*	**1.7**	1.0-2.4	*157*
2008	**21.3**	18.3-24.3	*372*	**2.9**	2.5-3.2	372*	**14.1**	11.3-16.9	*168*	**1.7**	1.3-2.2	*174*

Po = period from symptom onset until notification

Pd = period from laboratory diagnosis until notification

* p < 0.05 for 2008 versus 2003 and 2004

n = number of notifications

### Notification procedures

All 31 MHS in the Netherlands returned the questionnaire. Of these, 25 MHS had agreements with physicians to authorise direct laboratory reporting by one or more laboratories in their region. With regards methods of reporting by physicians and laboratories, five MHS received most reports by post, and 12 MHS received them largely by fax. Others reported a combination of these two methods, with telephone sometimes used in case of urgency; seven MHS received most laboratory reports by e-mail. For all the MHS, reports from physicians came most often by post or by telephone and rarely by e-mail.

Comparing the timeliness of a total of 1533 HBV infection reports to MHS in 2008, with respect to the presence or absence of physician laboratory agreements, MHS with agreements showed a significant reduction in notification time by 5.3 days (p < 0.01; 95% CI 1.7-8.9 days), compared to MHS without agreements.

MHS, which received most reports by fax, showed an average improvement in notification time of 3.3 days (p < 0.05; 95% CI 0.5-6.1 days) compared to MHS which received reports by post. E-mail was slower than fax, though not significantly, and showed no significant improvement compared to post.

Most MHS received about five to ten report cards per week. Only one MHS indicated that it still received 10-20 report cards per week from physicians, and its average Pd of notifications showed a significant delay of 19,1 days compared to the other MHS with an average Pd of 7,3 days. No MHS received more than 20 report cards per week.

No MHS received a majority of notifications by e-mail. Most MHS that used e-mail had encountered problems with the security of their inhouse electronic mail system. Moreover, to protect patient privacy, an e-mail report would often contain a minimum of information, requiring a public health officer to spend time contacting the laboratory or physician to complete the report.

## Discussion

This is the first study conducted in the Netherlands on the timeliness of infectious disease reporting at the national level. Our key measures were the intervals between onset of symptoms and MHS notification, and between laboratory diagnosis and notification.

It is preferable for notification to the MHS to occur within the incubation period to prevent transmission leading to secondary cases. The interval after diagnosis can be influenced by faster reporting procedures.

We found that even in this small and highly industrialised country, during the period studied, a considerable number of infectious disease cases were not reported to the Municipal Health Service (MHS) within the time frame of two incubation periods, and many cases were reported more than three days after laboratory confirmation of diagnosis. This striking delay in reporting leads to considerable delay of response measures by the MHS and the National Institute for Public Health and the Environment (RIVM). Differences in timeliness of reporting for each disease cannot only be attributed to differences in incubation periods. Cases of measles and typhoid fever, as well as cases of meningococcal disease and EHEC/STEC infections, show different percentages, despite their incubation periods being very similar. We find that each disease has specific attributes that have to be analysed for each step in the surveillance process in order to identify reasons for delays and to find options for improvement [4,7].

Shigellosis and EHEC/STEC infections have short incubation periods and require time-consuming laboratory testing for diagnosis. These two disease categories consequently show remarkable delays in reporting; for shigellosis, we found even higher percentages of delay than were found in other studies [6-8]. We hypothesise that shigellosis patients in the Netherlands present to their general practitioner at a later stage of the disease than patients in other countries, and that laboratory diagnosis is likewise requested at a later stage. However, we were not able to investigate these hypotheses. We did find that physicians and laboratories are not aware of the importance of rapidly reporting these cases, leading to an increase in the percentage of shigellosis and EHEC/STEC infections that are reported more than three days after diagnosis. This may be a cause for concern as has been found in other studies [18]. Public health response measures, such as improving hygiene and implementing fast exclusion policies in schools or institutions, are sometimes urgently required in order to avoid outbreaks of these two diseases. We must therefore conclude that Dutch surveillance is not sufficient with regard to these infections, and that additional approaches to control, such as public education on hygiene and raised awareness of urgency among physicians, must be considered.

The severity of illness of meningococcal disease generally creates a sense of urgency for the diagnosing physician, which no doubt extends to reporting. Chemoprophylaxis, preferably administered within 24 hours after identification of the index case, is the primary means for preventing meningococcosis, and the ability of health services to identify contacts in time, depends largely on timeliness of reporting. Nevertheless, we found that 13.3% of cases of meningococcosis are not reported within two incubation periods, a total of seven days. Other studies have likewise found suboptimal timeliness of reporting cases of this disease [9,10]. Optimising Pd, as shown in Table 1, by immediate laboratory reporting, can make an essential improvement and should be applied.

Using the time interval corrected for infectiousness before disease onset (Ic), we found that even diseases with longer incubation periods (measles, HAV infection) have a considerable percentage of cases notified after one Ic. Although overall vaccination coverage for measles is high in the Netherlands, unvaccinated subgroups persist. It is therefore cause for concern that 51.5% of cases are not notified within one Ic. For measles, a rapid vaccination campaign for contacts of infectious cases is essential for controlling an outbreak.

HAV infections occasionally cause outbreaks in the Netherlands in child day-care centres, schools, or other institutions. Most of these outbreaks are initiated by children of immigrants, who become infected when travelling to their parents' home countries [19]. The effectiveness of active or passive immunisation of contacts in preventing illness depends on the time elapsed after HAV exposure. Since HAV infections have a long incubation period, it is acceptable that 90.3% of cases are reported within one incubation period. However, the percentage of cases reported within one Ic should be improved.

Given that direct laboratory reporting can reduce delays, Dutch legislation was introduced in December 2008 to make laboratory reporting mandatory in addition to the mandatory reporting by physicians. This could considerably improve the reporting speed for MHS lacking physician agreements, an assumption that we will analyse in the coming years.

The findings of the present study are subject to some limitations. Several MHS merged during the study period, causing some loss of data from former MHS. Also, many MHS, especially those serving large cities, receive reports from several laboratories but do not have physician agreements covering all of them, impeding a comparison of laboratories. However, our findings are supported by other studies in the Netherlands that deal with physician-laboratory-MHS agreements [14].

Many cases of measles are reported solely on the basis of epidemiological criteria (i.e. a clinical case definition combined with known exposure to someone with a laboratory confirmed diagnosis) and need no laboratory confirmation. As there is no laboratory-related delay in such cases, the over all median Po may be shorter for measles. For the other diseases we studied, the proportion of cases reported solely on the basis of epidemiological criteria, is negligible.

In assessing the report cards posted by physicians to their MHS, we used the absolute number of cards and did not correct for variation in MHS region size. However, the MHS that received the most cards is not situated in one of the largest regions, and its total number of notifications is in the mid-range.

For all six of the infectious diseases considered, we have demonstrated how much timeliness in notification could be improved if MHS were to receive reports from physicians on the day of laboratory diagnosis (assuming that Pd is zero), thereby shortening Po. Although the Pd will not be zero for cases diagnosed on weekend days, this does not detract from the possible improvement during working days, when most cases are diagnosed. Agreements between physicians and laboratories that allow direct laboratory reporting can reduce delay by several days, and fast communication methods such as fax or telephone are preferable to post.

However, the results of the means of the Po per year, did not show an improvement for shigellosis or HAV infection, while the means of the Pd decreased by one day (statistically significant only for shigellosis). Apparently, a one day decrease in the Pd is not significant enough to result in a difference in the means of the total time delay. Nonetheless, the median values and percentages of infections reported within the incubation period do show a clear improvement by direct reporting.

Interestingly, the use of e-mail by reporting physicians or laboratories does not seem to accelerate the process, largely due to security problems.

Some countries have experienced improvement of timeliness through direct and automated electronic laboratory reporting (ELR) [20-24]. Such systems allow physicians to report over the internet (using web forms), and data can even be imported from the laboratory computer systems, eliminating manual data entry. Reporting methods and procedures should be optimised in the Netherlands, for example, by developing more secure e-mail approaches, adjusted web forms for reporting, or automated ELR.

In this study we chose to compare median incubation periods with median notification times as a measure of timeliness. However, this comparison has two major shortcomings. One is that incubation time measures the time between infection and appearance of symptoms, but not the time to infectiousness and further transmission. With diseases in which onward transmission occurs before symptoms appear, the latent period is a better measure of timeliness. Therefore we introduced the period Ic for HAV infection and measles as measure of timeliness instead of the incubation period. It follows that the optimal way to define timeliness would be to base it on the generation interval, i.e. the time between infection of the index case and infection of its secondary case. However, as the generation interval is difficult to observe the serial interval is usually used, i.e. the expected time between symptom onset in the index case and symptom onset in its secondary case [11,17].

The second problem in measuring timeliness is that intervals for notification and latent periods are not fixed but distributed, possibly with a large variance.

**Figure 3:**
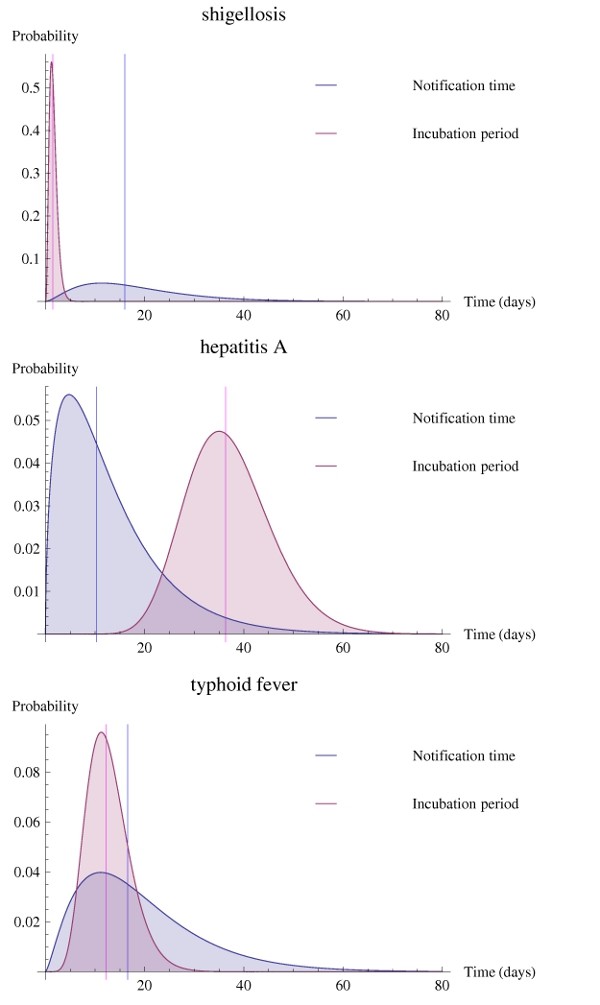
**Distributions of the incubation period (pink curves) and reporting time (grey curves), with their median values (lines), of shigellosis, hepatitis A virus infection, and typhoid fever cases**.

In Figure 3, it is readily noticeable that for shigellosis, a disease with a very short incubation time (low variance), the distribution of the reporting time lies far beyond this incubation interval and has a large variance. In contrast, for HAV infection the curves are more alike, showing a large proportion of cases within the incubation period.

A comprehensive analysis requires taking into account the distribution of notification time intervals and comparing it to the distribution of generation times. We are currently working on an analysis of timeliness based on the distributional properties of the relevant time intervals.

## Conclusions

In recent years in the Netherlands, a disappointingly large number infectious disease cases have not been reported within the incubation period, especially when the incubation period is short and laboratory testing is time-consuming, resulting in a considerable delay of response measures. For shigellosis and EHEC/STEC infections, additional control measures may therefore be necessary.

Even for diseases with long incubation periods, such as measles and HAV infection, a considerable percentage has been reported after a time interval corrected for the period of infectiousness before disease onset (Ic).

Delays in patient presentation or in performance of laboratory testing can cause unavoidable delays in MHS notification. However, delay after laboratory diagnosis can be minimised by optimising reporting procedures and using fast communication methods. We show that in the Netherlands, the use of adjusted web forms for reporting or of automated laboratory reporting systems have yet to be explored.

Whatever the systems used, evaluating the time intervals of reporting for each infectious disease must be an ongoing process, and the development of international standardised methods to measure timeliness needs to be promoted.

The use of intervals such as latent period and generation time and the distributions of those intervals may allow better study of the concept of timeliness in infectious disease surveillance systems.

## Competing interests

The authors declare that they have no competing interests.

## Authors' contributions

ER carried out the study in the context of the Training Program for Medical Specialists in Public Health at the Netherlands School of Public & Occupational Health, performed statistical analysis, and drafted the manuscript. CS and JvS supervised and participated in the design of the study and helped to draft the manuscript. MK has been involved in drafting the manuscript and revising it critically for important intellectual content. All authors read and approved the final manuscript.

## Pre-publication history

The pre-publication history for this paper can be accessed here:

http://www.biomedcentral.com/1471-2458/11/409/prepub
